# CAR T-Cells Depend on the Coupling of NADH Oxidation with ATP Production

**DOI:** 10.3390/cells10092334

**Published:** 2021-09-06

**Authors:** Juan C. Garcia-Canaveras, David Heo, Sophie Trefely, John Leferovich, Chong Xu, Benjamin I. Philipson, Saba Ghassemi, Michael C. Milone, Edmund K. Moon, Nathaniel W. Snyder, Carl H. June, Joshua D. Rabinowitz, Roddy S. O’Connor

**Affiliations:** 1 Department of Chemistry, Princeton University, Princeton, NJ 08544, USA; juancarlos_garcia@iislafe.es; 2 Lewis-Singer Institute for Integrative Genomics, Princeton University, Princeton, NJ 08544, USA; 3 Center for Cellular Immunotherapies, Perelman School of Medicine at the University of Pennsylvania, Philadelphia, PA 19104, USA; davidheo153@gmail.com (D.H.); jleferov@pennmedicine.upenn.edu (J.L.); chongxu@pennmedicine.upenn.edu (C.X.); bphili@pennmedicine.upenn.edu (B.I.P.); ghassemi@pennmedicine.upenn.edu (S.G.); michael.milone@pennmedicine.upenn.edu (M.C.M.); cjune@upenn.edu (C.H.J.); 4 Department of Pathology and Laboratory Medicine, Perelman School of Medicine of the University of Pennsylvania, Philadelphia, PA 19104, USA; 5 A.J. Drexel Autism Institute, Drexel University, Philadelphia, PA 19140, USA; strefely@pennmedicine.upenn.edu (S.T.); natewsnyder@temple.edu (N.W.S.); 6 The Parker Institute for Cancer Immunotherapy, Perelman School of Medicine, University of Pennsylvania, Philadelphia, PA 19104, USA; 7 Department of Medicine, Perelman School of Medicine, University of Pennsylvania, Philadelphia, PA 19140, USA; edmund.moon@pennmedicine.upenn.edu; 8 Division of Pulmonary, Allergy, and Critical Care, Perelman School of Medicine at the University of Pennsylvania, Philadelphia, PA 19104, USA; 9 Penn SRP center, Center of Excellence in Environmental Toxicology and Department of Systems Pharmacology and Translational Therapeutics at the University of Pennsylvania, Philadelphia, PA 19104, USA

**Keywords:** armor CAR T-cells, *Lactobacillus brevis* NADH oxidase, LDHA

## Abstract

The metabolic milieu of solid tumors provides a barrier to chimeric antigen receptor (CAR) T-cell therapies. Excessive lactate or hypoxia suppresses T-cell growth, through mechanisms including NADH buildup and the depletion of oxidized metabolites. NADH is converted into NAD^+^ by the enzyme *Lactobacillus brevis* NADH Oxidase (*LbNOX*), which mimics the oxidative function of the electron transport chain without generating ATP. Here we determine if *LbNOX* promotes human CAR T-cell metabolic activity and antitumor efficacy. CAR T-cells expressing *LbNOX* have enhanced oxygen as well as lactate consumption and increased pyruvate production. *LbNOX* renders CAR T-cells resilient to lactate dehydrogenase inhibition. But in vivo in a model of mesothelioma, CAR T-cell’s expressing *LbNOX* showed no increased antitumor efficacy over control CAR T-cells. We hypothesize that T cells in hostile environments face dual metabolic stressors of excessive NADH and insufficient ATP production. Accordingly, futile T-cell NADH oxidation by *LbNOX* is insufficient to promote tumor clearance.

## 1. Introduction

The success of CAR T-cells in acute and chronic leukemia highlights their therapeutic promise against cancer. CARs are synthetic receptors that control antigen specificity, signal transduction, and effector function in a single polypeptide. Previously, we showed that CAR design has a profound influence on cellular metabolism; CARs expressing CD28 signaling domains yield glycolytic, effector T-cells whereas CARs expressing 4-1BB promote the development of mitochondrial-enriched, memory T cells [1]. Implicit in these earlier discoveries is that T cell metabolism is not fixed and can be dynamically modified to suit the target environment.

Extending the therapeutic impact of CD28 or 4-1BB-based CAR T-cells to solid tumors is a significant priority for the clinical domain. Often T-cells can effectively penetrate solid tumors and undergo antigen-specific stimulation; however, their ability to form cytolytic effector cells is impaired [2,3]. Metabolic checkpoints including nutrient depletion and oxygen deprivation contribute to T-cell dysfunction in solid tumors. Functional competence is restored as T-cells evacuate tumor regions and colonize oxygen-rich, nutrient-rich environments, such as non-draining lymph nodes [4] or even standard tissue culture environments [2,5]. These findings underscore the need for novel strategies that sustain CAR T-cell metabolic function in harsh environments.

The exact mechanism(s) limiting T-cell metabolism in solid tumors is unknown. Mitochondrial function as measured by an ability to synthesize new mitochondria in response to extrinsic stimuli and undergo high rates of oxidative phosphorylation for energy production is severely impaired in T cells traversing hypoxic tumors [4]. Conditioning agents that support T-cell mitochondrial biogenesis [6], and small molecules that selectively impair oxidative phosphorylation in tumor cells [7] enhance the antitumor function of T-cells in melanoma. Reducing competition for glucose by genetically depleting tumor cell (but not T-cell) glucose transporter expression yielded less benefit. Thus, energy deficits from limited substrate availability may not always be the principal metabolic reason underlying T-cell hypofunction in cancer.

T-cell redox imbalances as measured by elevated NADH/NAD^+^ ratios have also been observed in mouse models of melanoma [8]. All cells rely on the reducing power of NADH to support ATP synthesis in the electron transport chain (ETC). Reductive stress, as measured by excess NADH production, can occur when the ETC is impaired in high lactate environments and hypoxia. Reductive stress can also impact cytoplasmic metabolism, suppressing both glycolysis and serine production [9]. To date no study has addressed how reductive stress impacts antitumor function, particularly in activated CAR T-cells undergoing high rates of Warburg metabolism in hostile environments.

For these reasons, we devised an approach to “arm” T-cells with an enzyme that restores redox balance whilst simultaneously catalyzing the conversion of lactate to pyruvate. *Lactobacillus brevis* NADH oxidase (*LbNOX*) fulfills this dual role. The bacterial enzyme *LbNOX* was effectively repurposed as a genetic tool to regulate redox status in HeLa cells [10]. A mitochondrial form of *LbNOX* normalized NADH/NAD^+^ ratios, decreased reductive stress, and rescued proliferative defects in fibroblasts treated with an inhibitor to complex 1 of the ETC [11]. A more recent study provided evidence that *LbNOX* restored redox balance in chronically stimulated mouse T-cells [8]. Collectively, these data support our hypothesis that heterologous *LbNOX* expression will restore functional competence to CAR T-cells traversing lactate-rich, hypoxic tumor environments.

Applying *LbNOX* to our cell culture and xenograft models allowed us to isolate the impact of lactate-induced reductive stress, independent of energy production, on the metabolic attributes and antitumor function of CAR T-cells. Understanding the relative importance of redox balance versus energy deficits is important to design metabolic strategies to advance CAR T-cell therapies against cancer. We found that *LbNOX*-expressing CAR T-cells have greater total oxygen consumption relative to control CAR T-cells, are strategically poised to oxidize lactate as a fuel in support of TCA cycle anaplerosis and withstand ETC inhibition. Despite these metabolic attributes, *LbNOX* expressing CAR T-cells displayed inferior tumor control in a xenograft model of mesothelioma, suggesting that T-cells depend on the coupling of NADH oxidation with ATP production by mitochondrial respiration.

## 2. Materials and Methods

### 2.1. Cell Culture

Primary human leukocytes (PBLs) from healthy male and female volunteers, averaging 34 years of age, were collected at the University of Pennsylvania’s Apheresis Unit. Informed consent was obtained from all participants prior to collection. All methods and experimental procedures were approved by the University of Pennsylvania Institutional Review Board (Protocol #11705906). T-cells were purified at the University’s Human Immunology Core by negative selection using the RosetteSep T-cell enrichment cocktail. Following isolation, T-cells were cultured in growth medium (GM) comprising RPMI 1640 (Lonza, Basel, Switzerland) supplemented with 10% FBS (Hyclone, Logan, UT, USA), 10 mM HEPES, 2mM L-glutamine, 100 U/mL penicillin G, and 100 µg/mL streptomycin. For T-cell activation, 4.5 µm Dynabeads containing immobilized anti-human CD3 and anti-human CD28 (Life Technologies, Carlsbad, CA, USA) were used at a ratio of 3 beads to 1 cell. T cells were maintained in culture at a concentration of 0.8–1.0 × 10^6^ cells/mL through regular counting by flow cytometry using CountBright beads (BD Biosciences, Franklin Lakes, NJ, USA), a viability marker (Viaprobe) and mAbs to either human CD4 or CD8 as described O’Connor et al. [12]. Lymphocytes were cultured at 37 °C, 20% O_2_, and 95% humidity with 5% CO_2_ unless otherwise stated.

A patient-derived human mesothelioma cell line (EM-meso), genetically engineered to stably express mesothelin and click beetle green (CBG) luciferase, has been previously described [2].

To isolate murine CD8+ T cells, spleens were harvested, and single-cell suspensions prepared by manual disruption and passage through a 70 mm cell strainer in PBS supplemented with 0.5% BSA and 2 mM EDTA. After red blood cell lysis, naive CD8+ T cells were purified by magnetic bead separation using commercially available kits following vendor instructions (Naive CD8a+T Cell Isolation Kit, mouse, Miltenyi Biotec Inc., Germany). Murine T-Cells were cultured in complete RPMI media (supplemented with 10% FBS, 100U/mL penicillin, 100 mg/mL streptomycin, and 50 mM 2-mercaptoethanol). For activation, T-cells were stimulated for 48 h with plate-bound anti-CD3 (10 mg/mL) and anti-CD28 (5 mg/mL) in complete media supplemented with recombinant IL-2 (100 U/mL). Cells were maintained in complete RPMI media supplemented with 100 U/mL recombinant IL-2. Metabolomics experiments were performed at day 4–5 post-activation.

### 2.2. LbNOX and mitoLbNOX Lentiviral Plasmid Construction

pTRPE is a bicistronic lentiviral vector containing a T2A ribosomal skipping sequence that separates two unique coding sequences that are co-translated as separate proteins. pTRPE_eGFP contains the open reading frame for eGFP upstream of T2A permitting an accurate measurement of lentiviral-mediated gene delivery by flow cytometry. The second gene sequence is positioned within AVR11 and Sal1 restriction sites. Expression plasmids for *LbNOX* and mito-*LbNOX* that have been codon-optimized for mammalian cells were kindly provided by Dr. Vamsi Mootha. Using standard molecular biology techniques, a 1.417kb cDNA insert was PCR-amplified using PUC57-Lb LBNOX plasmid as a template with forward (5′-CGT**CCTAGGATG**AAGGTCACCGTGGTCGGA-3′) and reverse primers (5′-CGT**GTCGAC**TTACTTGTCATCGTCATCC-3′) containing built-in AVR11 (underlined) and Sal1 (underlined) restriction sites. The purified PCR product and pTRPE_eGFP-T2A were digested with the relevant enzymes (NEB), gel purified, and ligated at a 3:1 insert:vector ratio using T4 DNA ligase to create a pTRPE_eGFP-T2A_*LbNOX* lentiviral plasmid. Similarly, a 1.484kb mitochondrial targeted LBNOX (mito*LbNOX*) cDNA insert was PCR amplified using PUC57- Mito*LbNOX* as a template with forward (5′-AGC**CCTAGG**ATGCTCGCTACAAGGGTCTTTA-3′) and reverse primers (5′-CGT**GTCGAC**TTACTTGTCATCGTCATCC-3′) containing built–in Avr11 (underlined) and Sal1(underlined) restriction sites. The purified PCR product and pTRPE_eGFP-T2A were digested with the relevant enzymes (NEB), gel purified, and ligated at a 3:1 insert:vector ratio using T4 DNA ligase to create a pTRPE_eGFP-T2A_mito*LbNOX* lentiviral plasmid. In assessments of cell proliferation, enumeration was performed using bead-based counting methods following gating on GFP+ cells.

### 2.3. Lentiviral Production

The lentiviral vector pTRPE encodes discrete gene products under the transcriptional control of EF-1 α. Lentiviral supernatants were generated by transient transfection of 293-T cells with pTRPE. Then, 293-T cells were initially seeded in T150 flasks and grown to 80% confluence in 25 mL of culture medium (RPMI1640), and 90 µL Lipofectamine 2000 DNA transfection reagent was combined with 7 µg pCL-VSVG, 18 µg pRSV-REV, and 18 µg of pGAG-POL (Nature Technology Corporation, Lincoln, NE, USA), as well as 15 µg of pTRPE. This mixture was incubated at room temperature for 15 min. DNA-lipofectamine complexes were then added to the 293-T cells. After 24 h, infectious supernatants were sterile filtered through a 0.45-μm syringe tip cellulose acetate filter and collected in a 50 mL conical tube. To pellet the lentivirus, the supernatant was spun in a Thermo Fisher Scientific Centrifuge (LYNX 4000) at 18,000 RCF, overnight, at 4 °C. The supernatant was removed, and the lentiviral pellet was resuspended in 1.6 mL of culture medium, aliquoted, and stored at −80 °C. The mesothelin-specific CAR lentiviral plasmid was previously described [5] and contains the SS1 scFv, CD8α hinge, and CD8α transmembrane domain linked to the CD28 costimulatory domain and the CD3ζ signaling domain under the transcriptional control of an EF1α promoter.

### 2.4. Lentiviral Infection

Primary human T-cells were activated with Dynabeads as described above. Furthermore, 24 h after activation, T cells were seeded at 100,000 cells/well at a concentration of 1 × 10^6^ cells/mL in a 96-well culture dish. Serial dilutions of lentiviral supernatant over a range of 1:3, 1:9, 1:27, 1:81, 1:243, and 1:729 were performed. Transduced T-cells were grown for 72 h to ensure optimal gene expression before comparing transduction efficiencies. The percentage of GFP+ cells was determined by flow cytometry, and the corresponding titer was calculated as the number of transforming units/mL. The titers for pTRPE_eGFP-T2A_*LbNOX* and pTRPE_eGFP-T2A_Mito*LbNOX* viral supernatants were 34.2 × 10^6^ and 35.5 × 10^6^ TU/mL, respectively. T cells were infected with lentiviral vectors at multiplicities of infection from 3–5. Titers for the SS1 CAR lentivirus were 58.3 × 10^6^ TU/mL.

### 2.5. LDH Inhibition

LDH inhibitor NCGC00420737 was obtained from the NCI Experimental Therapeutics (NExT) Project team located in Bethedsa, MD, USA [13]. A 10 mg aliquot of compound NCGC00420737 was added to 250 µL of 0.1M NaOH. This solution was sonicated for 10 min. Then, 750 µL of PBS was added and the solution was sonicated for an additional 10 min. Finally, the pH was adjusted to 7.5, and the solution was passed through a 0.2 μM syringe filter.

### 2.6. Mitochondrial Respiratory Features as a Function of LBNOX Expression

Mitochondrial function was assessed using an extracellular flux analyzer (Agilent/Seahorse Bioscience, Santa Clara, CA, USA). Individual wells of an XF96 cell culture microplate were coated with CellTak in accordance with the manufacturer’s instructions. The matrix was adsorped overnight at 37 °C, aspirated, air dried, and stored at 4 °C until use. Following overnight stimulation with Dynabeads, T-cells were *LbNOX*, and expanded for three days. To assay mitochondrial function, T cells were centrifuged at 1200× *g* for 5 min. Cell pellets were re–suspended in XF assay medium (non–buffered RPMI 1640) containing 10 mM glucose, 2 mM L-glutamine, and 5 mM HEPES. T-cells were seeded at 0.2 × 10^6^ cell/well. During instrument calibration, the microplate was centrifuged at 1000× *g* for 3 min and switched to a CO_2_-free, 37 °C incubator for 30 min. Cellular oxygen consumption rates (OCR) were measured under basal conditions and following treatment with 20 mM sodium–L–lactate (MilliporeSigma St. Louis, MO, USA), 1.5 μM fluoro-carbonyl cyanide phenylhydrazone (FCCP), and 500 nM rotenone/antimycin A.

### 2.7. Extracellular Acidification as a Function of LDHA Inhibition

To assess lactic acid production in EM-meso cancer cells, 0.1 × 10^6^ EM-meso cells were seeded onto uncoated XF96 microplates. The following day, the cells were washed in PBS, and the medium was switched to the customized XF assay medium described above. During instrument calibration, the microplate was switched to a CO_2_–free 37 °C incubator for 30 min. Extracellular acidification rates (ECAR) were measured under basal conditions and following treatment with 10m M glucose, 1.3 µM oligomycin, varying concentrations of LDHi (5–50 µM), and 20 mM 2-deoxyglucose (2-DG).

### 2.8. Anti-Mesothelin 28ζ CAR T-Cell Cytolytic Function

T-cells were activated with Dynabeads as described above. Following overnight stimulation, activated T cells were co-infected with a 28**ζ** CAR against mesothelin and either a eGFP control lentiviral vector or a vector expressing *LbNOX*. Mock infected (nontransduced) T-cells were used as a control. Activated T-cells were then expanded for 9 days until restdown (Cell size: 340–400 fL). EM-meso target cells were seeded at overnight in a U-bottom 96-well plate at 10,000 cells/well. The following day, CAR T-cells were added at established effector:target cell ratios of 3:1. CAR T-cell mediated killing, as measured by a decrease in the luciferase signal generated by live target cells, was assessed at 24 h. Briefly, Luciferin was added at a final concentration of 150 µg/mL per well. Luminescence was measured after 10-min incubation using the Envision (PerkinElmer, Waltham, MA, USA) plate reader, and luciferase activity was expressed as relative luminescence units (RLUs). Note that target cells incubated in medium alone or treated with 1% SDS were used to calculate spontaneous cell death (RLU_spon_) or maximal cell death (RLU_max_), respectively. The percent specific lysis was calculated using the formula: % specific lysis = 100 × ([RLU_spon_ − RLU_test_]/[RLU_spon_ – RLU_max_]).

Mean luciferase activity (from at least 5 replicates) was calculated and compared across each treatment group. All data are presented as mean ± SEM.

### 2.9. In Vivo Xenograft Studies

A xenograft model was used in this study as previously reported [5]. Briefly, 6–10-week-old NOD-SCID γ_c_^−/−^ (NSG) mice, which lack an adaptive immune system, were obtained from Jackson Laboratories (Bar Harbor, ME, USA) or bred in-house under a protocol approved by the Institutional Animal Care and Use Committees of the University of Pennsylvania. Animals were assigned in all experiments to treatment/control groups using a randomized approach. Animals were injected subcutaneously with 5 × 10^6^ Em-meso tumor cells in 0.1 mL sterile PBS. After tumors reached >200 µM^2^, mice were randomized to each treatment group. Anti-mesothelin CAR T-cells co-expressing either *LbNOX* or eGFP and non-transduced NTD human T-cells were injected I.V. at the indicated dose in 100 µL of sterile PBS. Oral gavage (Ldhi vs. PBS vehicle) was performed (75 mg/kg twice weekly) and three times weekly (days 52–59) using an 18–20 G needle. Tumor size was measured biweekly using digital calipers.

### 2.10. Isotope Labeling

For lactate-labeled isotope experiments, T-cells were activated with Dynabeads as described above. Following overnight stimulation, the cells were expanded for 6 days with regular counting and feeding on alternate days. The medium was then switched to RPMI 1640, conditioned with 10% dialyzed FBS (Life Technologies, Carlsbad, CA, USA), and supplemented with 20 mM U-13C-Lactate (MilliporeSigma, MA, USA) for 1 h.

### 2.11. Short-Chain Acyl-CoA Extraction

Extractions were performed as described previously [14]. Briefly, lymphocytes were centrifuged at 1200× rcf for 5 min. Cell pellets were resuspended in 750 µL of ice-cold 10% trichloroacetic acid and pulse-sonicated using a sonic dismembrator (Fisher Scientific, Hampton, NH, USA). The samples were centrifuged at 15,000× rcf for 15 min, and the supernatants were purified by solid phase extraction. Briefly, Oasis HLB 1-mL (30 mg) solid-phase extraction columns were conditioned with 1 mL methanol, followed by 1 mL of H_2_O. The supernatants were applied to the column and washed with 1 mL of H_2_O. The analytes were eluted in methanol containing 25 mM ammonium acetate. The eluates were dried overnight in N_2_ gas and resuspended in 50 µL of 5% 5-sulfosalicylic acid, and 10 µL injections were applied in LC/ESI/MS/MS analysis.

### 2.12. Metabolite Extraction from Murine T-Cells

RPMI-1640 media without glucose and glutamine was supplemented with 10% dialyzed FBS, 100 U/mL penicillin, 100 mg/mL streptomycin, 50 mM 2-mercaptoethanol, and 100 U/mL recombinant IL-2. For 13C-glucose incubation, it was supplemented with 11 mM U-13C-glucose and 2 mM glutamine; for 13C-glutamine incubation, with 11 mM glucose and 2 mM U-13C-glutamine; and for 13C-lactate incubation, with 11 mM glucose, 2 mM glutamine, and 20 mM U-13C-lactate.

Cells were seeded at 106 cells/mL and incubated for 24 h. They were then transferred to 1.5 mL Eppendorf tubes and pelleted (3 min, 500× *g*, RT). Media was removed by aspiration, and 500 μL of PBS was added. Then, cells were pelleted (30 s, 6000× *g*, RT), PBS removed by aspiration, and metabolome extraction was performed by the addition of 100 μL of cold methanol:water (80:20). The extract was incubated at –20 °C for at least 30 min.

### 2.13. Analysis of Polar Metabolites in Murine T-Cells

After centrifugation (15 min, benchtop microfuge maximum speed, 4 °C), the clean supernatant was transferred to LC-MS vial for analysis. Samples were analyzed by reversed-phase ion-pairing chromatography coupled with negative-mode electrospray-ionization high-resolution MS on a stand-alone Orbitrap (ThermoFisher Exactive, Waltham, MA, USA) [15]. Data were analyzed using El-MAVEN software (Elucidata, Cambridge, MA, USA). Isotope labeling was corrected for natural 13C abundance [16].

### 2.14. Analysis of Fatty Acids in Murine T-Cells

Cell extracts were saponified fatty acids extracted and analyzed by reversed-phase ion-pairing chromatography coupled with negative-mode electrospray-ionization high-resolution MS on a stand-alone Orbitrap (ThermoFisher Exactive, Waltham, MA, USA) [17]. Data were analyzed using El-MAVEN software (Elucidata, Cambrdige, MA, USA). Isotope labeling was corrected for natural 13C abundance (Su et al. [16]). Relative contribution of the various carbon sources to fatty acid synthesis was calculated using R by fitting the data into a zero truncated binomial distribution.

## 3. Results

**Figure 1:**
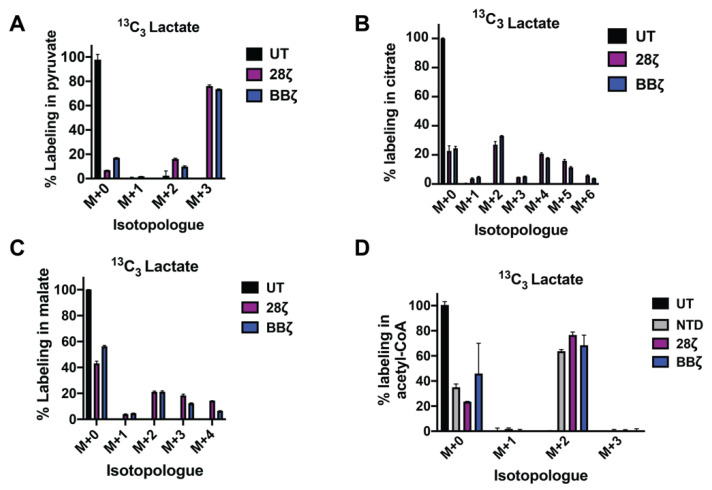
Lactate supports TCA cycle anaplerosis and short chain CoA synthesis in CAR T-cells. Activated CAR T-cells (day 7) were refed with RPMI medium, conditioned with 10% dialyzed FBS, and supplemented with 20 mM ^13^C_3_ lactate for 1 h. Mass isotopomer data for pyruvate (**A**) and citrate (**B**) are shown. Mean values ± S.E.M. from 3 technical replicates are shown. UT: untreated; 28ζ: T-cells lentivirally infected with a 28ζ CAR and treated with ^13^C_3_ lactate; BBζ: T-cells lentivirally infected with a 4-1BBζ CAR and treated with ^13^C_3_ lactate. (**C**,**D**) Activated T-cells (day 10) were refed with RPMI medium, conditioned with 10% dialyzed FBS, and supplemented with 20 mM ^13^C_3_ lactate overnight. Mass isotopomer data for malate and acetyl-CoA are shown. Mean values ± S.E.M. from 3 technical replicates are shown. NTD: nontransduced T-cells; 28ζ: T cells lentivirally infected with a 28ζ CAR and treated with ^13^C_3_ lactate; 28ζ NOX: T-cells lentivirally co-infected with a 28ζ CAR, as well as *Lactobacillus brevis* NADH Oxidase (*LbNOX*) and treated with ^13^C_3_ lactate BBζ: T-cells lentivirally infected with a 4-1BBζ CAR and treated with ^13^C_3_ lactate.

NADH and its oxidized derivative NAD^+^ support anabolic reactions in T-cells undergoing clonal expansion and differentiation. NAD^+^/NADH levels are highly regulated and exist in near-equilibrium with pyruvate and lactate. We used stable isotope labeling to trace the contribution of isotopically labeled (^13^C_3_) lactate to metabolic pathways in CAR T-cells. Activated T-cells were infected with a lentiviral CAR construct containing a mouse anti-human mesothelin scFv (SS1) linked to the human intracellular signaling domains CD28 and CD3ζ (Figure S1A). As CAR signaling profoundly influences T-cell metabolic activities [1] and clinical efficacy [18], we included CARs engineered with 4-1BB signaling domains (Figure S1B) in our analyses. At day seven of stimulation, coinciding with the mid-phase of logarithmic growth, we transferred activated T-cells to a cell culture medium conditioned with 10% dialyzed FBS and supplemented with ^13^C_3_-lactate. After one hour, we harvested the cells for LC-MS. We found that lactate labels ~75% of the intracellular pyruvate pool in both 28ζ and BBζ CAR T-cells, respectively (Figure 1B). An increase in the lactate/pyruvate ratio, derived from the addition of 20 mM ^13^C_3_-lactate, promotes, by mass action, pyruvate (M+3) labeling. These data show that extracellular lactate is a major source of intracellular pyruvate, even in glycolytic CAR T-cells. Similarly, in murine T-cells, lactate can replenish intracellular pyruvate as effectively as glucose (Figure S1B).

To determine if ^13^C_3_-lactate is effectively integrated into intracellular metabolic pathways downstream of pyruvate, we traced its contribution into the TCA cycle. We show that lactate provides an important source of TCA carbon, labeling 27% and 33% of the intracellular citrate pool as M+2 in 28ζ and BBζ CAR T-cells, respectively (Figure 1B). Moreover, 21% of malate was M+2 labeled in each cell type (Figure 1C). Thus, CAR T-cells are equipped to metabolize lactate.

Acetyl-CoA is an essential precursor for lipid, cholesterol, and isoprenoid synthesis. We investigated the contribution of lactate to acetyl-CoA pools following overnight culture of CAR T-cells with ^13^C_3_-lactate. CAR signaling modestly enhances the contribution of lactate to acetyl–CoA (Figure 1D). In murine T-cells, we show that lactate supports long chain fatty acid synthesis as effectively as glucose (Figure S1B), highlighting the ability of lactate to spare and/or replace glucose when necessary. These findings suggest that lactate can play a central role in CAR T-cell metabolism.

### 3.1. Arming T-Cells with Exogenous, Futile NADH Oxidation Capacity

**Figure 2:**
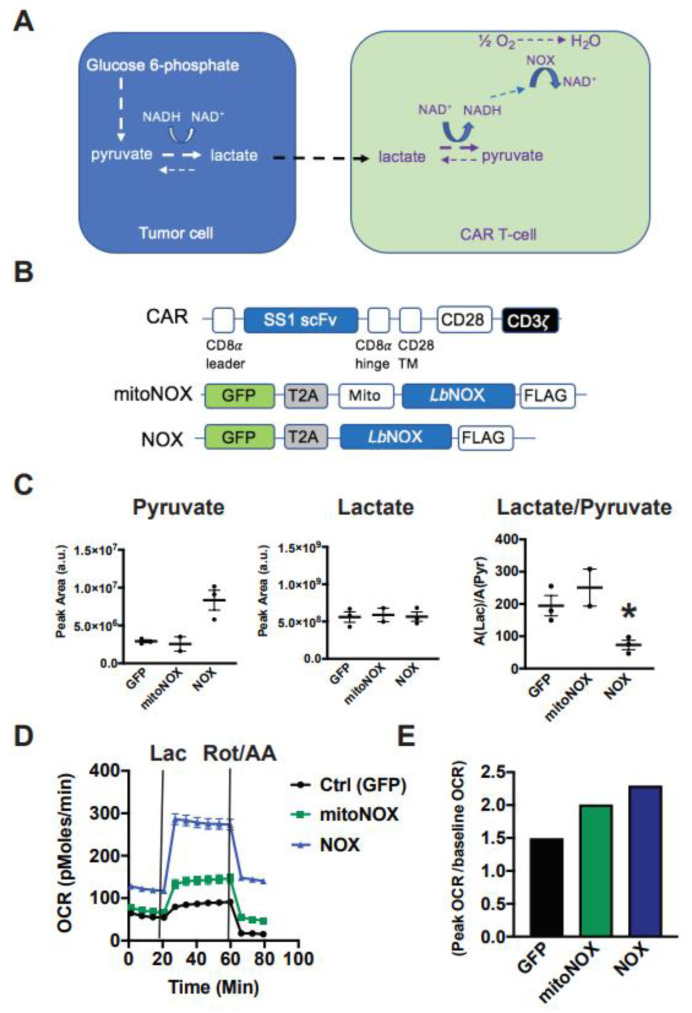
Cytoplasmic *Lactobacillus brevis* NADH Oxidase (*LbNOX*) reprograms T-cell metabolism towards lactate oxidation. (**A**) Our model proposing how *LbNOX* promotes lactate metabolism in CAR T-cells. Using NADH as a cofactor, *LbNOX* drives the vectorial flux of lactate to pyruvate in CAR T-cells. (**B**) Schematic representation of the mesothelin lentiviral CAR containing an SS1 single chain variable fragment (scFv), which is linked via a CD8α hinge, as well as a CD28 TM to the CD28 and CD3ζ intracellular signaling domains. TM, transmembrane. *LbNOX* is a bicistronic lentiviral construct containing the coding sequence for *LbNOX*, linked via T2A to the transduction marker GFP. A lentiviral construct encoding the mitochondrial (mito) NOX isoform is also shown. (**C**) After overnight stimulation with Dynabeads, activated T-cells were infected with either cytoplasmic or mito *LbNOX* lentivirus. Cellular supernatants were collected from NOX-expressing T-cells undergoing log-phase expansion. Lactate, as well as pyruvate levels, was compared by LC-MS. Mean values ± S.E.M are plotted with the horizontal bars representing the mean and each symbol representing a separate donor from independent experiments. The lactate/pyruvate ratio was significantly decreased in *LbNOX* relative to GFP (* *p* < 0.05). Statistical comparisons were performed using an unpaired Student’s *t* test. (**D**) After overnight stimulation with Dynabeads, activated T-cells were infected with either cytoplasmic or mito *LbNOX* lentiviral supernatants. These cells were expanded for 3 days and then transferred to bicarbonate-free XF assay medium. Metabolic parameters were measured by a Seahorse assay. Cellular oxygen consumption rates (OCR) were measured at baseline and following the serial addition of 20 mM lactate and 500 nM rotenone/antimycin A. Values are means ± S.E.M. from 7–8 replicates. Values are representative of 2 independent experiments. Baseline OCR levels were significantly increased in mitoNOX relative to GFP (*p* < 0.05); *LbNOX* relative to GFP (*p* < 0.05); and *LbNOX* relative to mitoNOX (*p* < 0.05). Lactate-stimulated OCR levels were significantly increased in mitoNOX relative to GFP (*p* < 0.05); *LbNOX* relative to GFP (*p* < 0.05); and *LbNOX* relative to mitoNOX (*p* < 0.05). Data were analyzed by a one-way ANOVA using a Holm–Sidak multiple comparison post hoc test. (**E**) Energy reserve (peak OCR/baseline OCR) as a function of *LbNOX* is shown. Values are calculated from the data illustrated in panel D.

Given its central role in oxidation/reduction reactions, an increase in lactate metabolism can have important consequences on the intracellular redox state. Lactate can be immune-suppressive by potentiating reductive stress in hypoxic environments. We engineered CAR T-cells with a bacterial-derived NADH-dependent oxidase to support the use of lactate as a fuel and dissipate redox gradients causing stress. Our central hypothesis is shown in Figure 2A. Using NADH as a cofactor, *LbNOX* catalyzes the transfer of free electrons to oxygen. In cells engineered to overexpress *LbNOX*, the production of NAD^+^ will push the pyruvate/lactate equilibrium towards pyruvate production and NADH replenishment by mass action. *LbNOX* catalyzes the reaction NADH + ½ O_2_ → NAD^+^ + H_2_O. Coupled to lactate dehydrogenase, the net reaction is lactate + ½ O_2_ → pyruvate + H_2_O. To study the potential benefit of *LbNOX* in primary human T-cells, we generated mitochondrial as well as cytoplasmic *LbNOX* lentiviral constructs (Figure 2B).

### 3.2. Cytoplasmic LbNOX Enhances T-Cell Oxidation

Previous studies showed that mitochondrial *LbNOX* improved oxidative function in fibroblasts [10]. To assess the impact of cytoplasmic versus mitochondrial *LbNOX* isoforms on T-cell redox status and substrate metabolism, we collected cellular supernatants from *LbNOX*-expressing T-cells undergoing log-phase expansion. *LbNOX* promotes NADH oxidation and can accordingly decrease the lactate/pyruvate ratio. Using LC-MS, we show that cytoplasmic *LbNOX* decreases the Lac/Pyr ratio by 63%, an effect largely driven by increased pyruvate, while mitochondrial *LbNOX* did not (Figure 2C). Consistent with these results, while both cytosolic and mitochondrial *LbNOX* increased oxygen consumption, cytosolic did so to a greater extent (Figure 2D).

We then measured the oxidative response to 20 mM lactate. Again, the respiratory response to lactate was accentuated in T-cells expressing the cytoplasmic rather than mitochondrial isoform of *LbNOX* (Figure 2E). Lactate increased OCR to 87, 141, and 278 pMoles O_2_/min in GFP, mitoNOX, and *LbNOX*-expressing T cells, respectively (* *p* < 0.05 for mitoNOX versus GFP; *p* < 0.05 for *LbNOX* relative to mitoNOX).

To simulate hypoxia, we measured the respiratory response to rotenone and antimycin A, inhibitors of the mitochondrial electron transport chain complex I and III, respectively. As seen in Figure 2D, *LbNOX*-expressing T-cells sustain higher rates of oxygen consumption (117% of their baseline OCR), whereas OCR levels in mitoNOX T-cells decreased to 70% of baseline, and OCR in control CAR T-cells diminished to 28% of baseline values. As the cytoplasmic isoform of *LbNOX* conferred superior metabolic attributes than its mitochondrial version, we pursued our studies with the cytoplasmic isoform only.

### 3.3. LbNOX Does Not Alter In Vitro CAR T-Cell Proliferation

**Figure 3:**
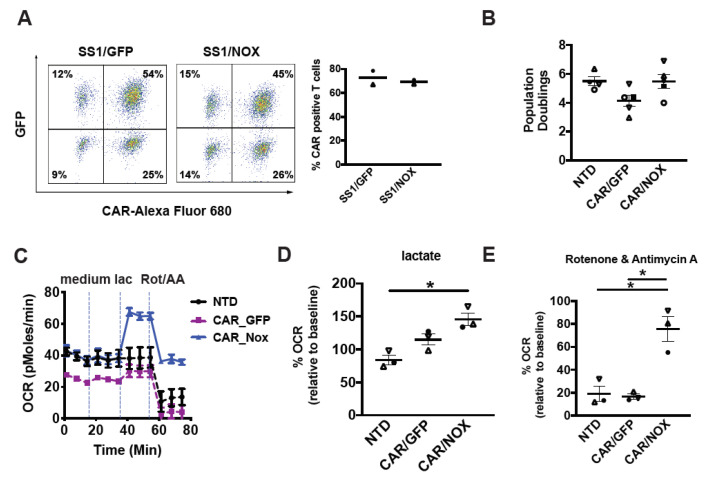
*Lactobacillus brevis* NADH Oxidase (*LbNOX*)-expressing CAR T-cells maintain high rates of oxygen consumption despite electron transport chain (ETC) inhibition**.** (**A**) After overnight stimulation with Dynabeads, T-cells were co-infected with a mesothelin-specific (SS1-28z) CAR and *LbNOX* lentiviral supernatants. These cells were expanded for 7 days. Surface SS1 CAR expression was measured by staining with a biotinylated goat anti-mouse IgG (H + L) followed by streptavidin (SA)-Alexa Fluor 680 labeling. *LbNOX* levels were simultaneously detected by GFP expression. CAR+ cells were defined as double positive for LbNOX (*Y*-axis) and APC (*X*-axis). Representative flow plots and mean frequencies ± SEM from two independent experiments with separate donors are shown (left and right panels, respectively). (**B**) After overnight stimulation with Dynabeads, activated T-cells were co-infected with a mesothelin-specific (28z) CAR and *LbNOX* lentiviral supernatants. Cell enumeration was performed every other day beginning on day 3 until the number of cells in the culture ceased increasing, and the mean cell volume was below 350 fL (day 10). The maximum number of population doublings is plotted with the horizontal bars representing the mean and each symbol representing a separate donor. (**C**) GFP vs. *LbNOX*-expressing CAR T-cells were expanded for 4 days and then transferred to bicarbonate free XF assay medium containing 10 mM glucose and 2 mM glutamine. Metabolic parameters were measured with a Seahorse assay. Cellular oxygen consumption rates (OCR) were measured at baseline, and following the serial addition of XF assay medium, 20 mM lactate and 500 nM rotenone/antimycin A. Representative data from 3 independent experiments with separate donors are shown. Values are means ± S.E.M from 6–8 replicates per assay. (**D**) OCR levels following lactate treatment are expressed as a percentage of baseline OCR. These are plotted with the horizontal bars representing the mean and each symbol representing a separate donor (*n* = 3). * *p* < 0.05 for CAR/NOX vs. NTD. (**E**) OCR levels following rotenone and antimycin A treatment are expressed as a percentage of baseline OCR (*n* = 3). * *p* < 0.05 for CAR/NOX versus NTD; * *p* < 0.05 for CAR/NOX vs. CAR/GFP. All data were analyzed by a one-way ANOVA using a Holm–Sidak multiple comparison post hoc test.

In the clinical sector, CAR T-cells are propagated over several days to increase their quantity prior to adoptive cell transfer. We set out to compare proliferative rates in CAR T-cells infected with *LbNOX* or control lentiviral constructs. We first confirmed equivalent CAR expression across experimental groups. As seen in Figure 3A, 77% of T-cells were double positive for CAR as well as the eGFP control plasmid. Similarly, 75% of T-cells were double positive for CAR as well as *LbNOX*. In line with clinical manufacturing protocols, these cells were expanded for 10 days until restdown. The effect of *LbNOX* on CAR T-cell proliferation and survival was assessed by flow cytometry at regular intervals during their proliferative phase. *LbNOX* had no adverse effect on T-cell proliferation or survival (Figure 3B).

### 3.4. LbNOX-Expressing CAR-T Cells Sustain Oxidative Metabolism despite ETC Inhibition

Given their individual impact on T-cell metabolic activity, we examined how *LbNOX* and CD28ζCAR co-expression impacts metabolism. With respect to CAR design, the CD28 signaling domain was preferred to 4-1BB as it confers superior effector function in several solid tumors tumor models including mesothelioma [5] and glioblastoma [19]. We co-infected activated T-cells with a mesothelin-specific 28ζCAR lentivirus along with *LbNOX* or GFP control lentivirus. After several days of expansion, the metabolic properties of *LbNOX* expressing CAR T-cells were tested by Seahorse assay. As seen in Figure 3C, baseline levels of oxygen consumption are higher in *LbNOX*-expressing CAR T-cells relative to control (GFP-expressing CAR T-cells). Interestingly, constitutive expression of a 28ζ CAR leads to lower baseline levels of oxidative metabolism relative to nontransduced controls. These findings corroborate our prior work demonstrating an increased emphasis of glycolysis over oxidative phosphorylation in 28ζ CAR T-cells [1]. As oxygen consumption increases in line with substrate metabolism, we measured OCR in cells treated with medium alone or medium containing 20 mM lactate. We show that *LbNOX* enhanced lactate-induced oxygen consumption by 141% (Figure 3D). As oxygen is critically limiting in solid tumors, we tested the ability of *LbNOX* CAR T-cells to maintain oxidative function in hypoxia-like conditions. To simulate the disruptive effects of hypoxia on respiratory function and oxidative metabolism, we treated CAR T-cells with rotenone and antimycin A. As seen in Figure 3E, *LbNOX*-expressing CAR T-cells maintain 91% of their baseline OCR (36.5 ± 0.6 pMoles/min), whereas OCR in control CAR T-cells decreases to 15% of baseline values (3.8 ± 0.4 pMoles/min).

### 3.5. LbNOX-Expressing CAR T-Cells Are Resilient to LDH Inhibition

**Figure 4:**
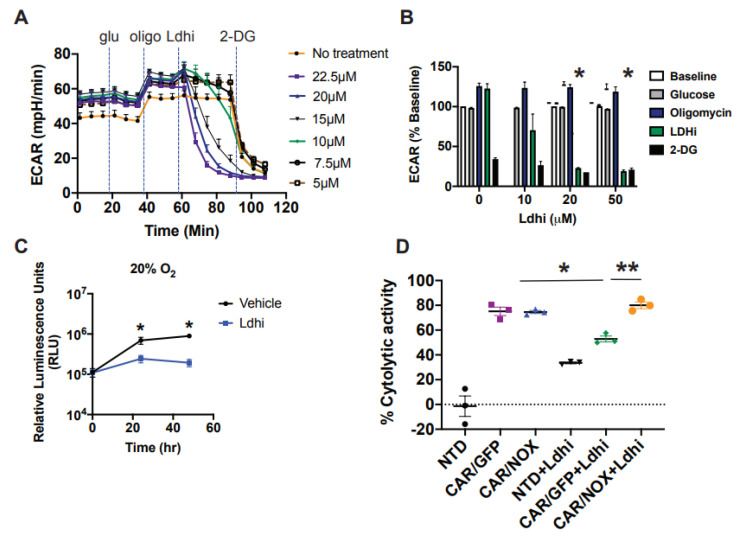
*Lactobacillus brevis* NADH Oxidase (*LbNOX*) rescues CAR T-cell cytotoxicity in cultures treated following Lactate Dehydrogenase (LDH) inhibition. (**A**,**B**) The metabolic properties of EM-meso cells were measured by a Seahorse assay. Extracellular acidification rates (ECAR) following the serial addition of 20 mM glucose, 1.5 uM oligomycin, Ldhi, and 20 mM 2-DG are shown in a representative Seahorse plot (left panel). Inhibiting LDH (Ldhi) reduces EM-meso cell glycolytic activity in a dose-dependent manner. Mean ± SEM values from multiple independent experiments with separate donors (*n* = 2–3) are shown (right panel). Ldhi ECAR values were measured 33 min following Ldhi injection and 20 μM Ldhi significantly reduced glycolytic activity relative to vehicle control (* *p* < 0.05). Statistical comparisons were compared using an unpaired Student’s *t* test. (**C**) EM-meso-CBG cells were seeded in triplicate in 96-well plates and treated with either the vehicle or 20 μM Ldhi. Their growth was measured by a luminescent assay. The mean ± S.E.M. values of 3 independent experiments are shown. * *p* < 0.05 for Ldhi vs. the vehicle at 24 h and 48 h. (**D**) The specific cytotoxicity of anti-mesothelin CAR T-cells was measured by luciferase-based killing assay. CAR T-cells were co-cultured with EM-meso cells at a 3:1 effector: target cell ratio for 22 h in medium conditioned with 20 μM Ldhi. The mean ± S.E.M. values of 3 independent experiments with separate donors are shown. * *p* < 0.05 for CAR/GFP vs. CAR/GFP+Ldhi; ** *p* < 0.05 for CAR/GFP+Ldhi vs. CAR/NOX+Ldhi. Data were analyzed by a two-way ANOVA using a Newman–Keuls multiple comparison post hoc test.

As *LbNOX* catalyzes the oxidation of NADH to NAD^+^, we tested its ability to rescue cytotoxicity in CAR T-cells treated with an LDH inhibitor. We reasoned that tumor cell glycolytic function could be selectively impaired by LDH inhibition if the corresponding CAR T-cells had a “built-in” mechanism to replenish NAD^+^. We used the LDH inhibitor NCGC00420737 to impair glycolytic function in EM-meso cells (Figure 4A,B). Inhibiting glycolysis with Ldhi significantly impeded tumor cell proliferation in vitro (Figure 4C). In cytotoxicity assays, *LbNOX*-expressing CAR T-cells retained complete functional competence despite LDH inhibition (Figure 4D).

**Figure 5:**
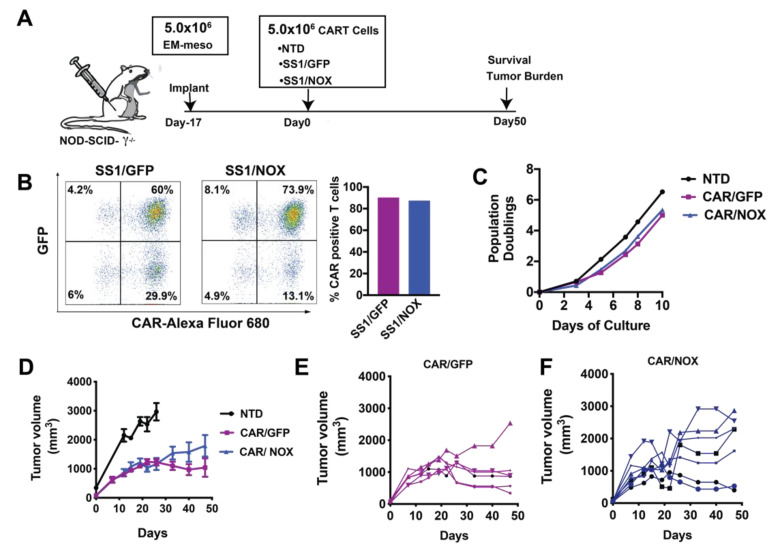
Investigating the antitumor function of *Lactobacillus brevis* NADH Oxidase (*LbNOX*)-expressing CAR T-cells. (**A**) Experimental layout including a schematic of the EM-meso xenograft model. (**B**) A total of 5 × 10^6^ EM-meso cells were injected subcutaneously into adult NSG mice. (**C**) Activated T-cells were co-infected with a mesothelin-specific (28ζ) CAR and *LbNOX* lentiviral supernatants, then expanded over 9 days. (**D**) Surface SS1 CAR expression was measured by staining with a biotinylated goat anti-mouse IgG (H+L) followed by streptavidin (SA) Alexa Fluor 680 labeling. *LbNOX* levels were simultaneously detected by GFP expression. CAR+ cells were defined as double positive for LbNOX (*Y*-axis) and Alexa Fluor 680 (*X*-axis). (**E**) Expansion curves showing the number of population doublings per cell over 10 days. Cell enumeration was performed every other day beginning on day 3 until the number of cells in the culture ceased increasing and the mean cell volume was below 350 fL. (**F**) A total of 5 × 10^6^ CAR+ T-cells were I.V. injected 17 days after tumor implantation (NTD: *n* = 4; CAR/GFP: *n* = 6; CAR/NOX: *n* = 7). Tumor volumes were measured by caliper at the indicated time-points as described in the Materials and Methods section. Values represent mean ± S.E.M. for each group. Tumor size (NTD vs. CAR/GFP vs. CAR/NOX) was compared by two-way ANOVA using a Holm–Sidak multiple comparison post hoc test. Tumor size was significantly less (*p* < 0.05) in all CAR expressing groups relative to the NTD control (analyzed at day 26, a threshold time-point when NTD were sacrificed). No statistical differences were observed across CAR treatment groups at the nadir of tumor development (day 40) or during the terminal phase of the study.

We next evaluated the antitumor function of *LbNOX* expressing CAR T-cells, with/without Ldhi using our well-established human xenograft model of mesothelioma. EM-meso xenografts establish an immune-suppressive tumor environment enriched with immune and metabolic checkpoints. Infused CAR T-cells effectively traffic to EM-meso tumors. Despite undergoing robust proliferation, their antitumor function is severely limited [5]. The experimental layout for testing the efficacy of anti-mesothelin CAR T-cells in this model is illustrated in Figure 5A. *LbNOX*-expressing, anti-mesothelin 28ζCAR-T cells were expanded over 10 days until restdown. CARs were expressed in 90% of T-cells in the control group. In the *LbNOX* group, 87% of T-cells expressed CAR. As seen in Figure 5B, 60% of T-cells were double positive for CAR as well as the GFP control plasmid. In the other experimental group, 74% of T-cells were double positive for CAR as well as *LbNOX*. To establish mesothelin xenografts, immunodeficient mice were subcutaneously injected with 5 × 10^6^ EM-meso tumor cells. After tumors reached 200 mm^3^, 5 × 10^6^ GFP or *LbNOX*-expressing CAR T-cells were injected intravenously. Tumor growth was monitored regularly over the next 50 days. As expected, EM-meso xenografts grew exponentially over time. Control T-cells (no CAR) had minimal impact on tumor cell growth (Figure 5D). Overall tumor volume was significantly reduced in tumor-bearing mice infused with CAR transduced T-cells (* *p* < 0.05 for CAR/GFP vs. NTD). Tumor control was incomplete but sustained through day 48 in this group (Figure 5D,E). *LbNOX*-expressing CAR T-cells also demonstrated significant tumor clearance (*p* < 0.05 for CAR/NOX vs. NTD); however, overall tumor burden remained higher than CAR T-cells alone (Figure 5D–F). Despite the potential for additive benefits from CAR T-directed cytotoxicity with Ldhi, we observed no additive benefit of Ldhi and CAR against tumor growth in our xenograft model of mesothelioma (Figure S3).

## 4. Discussion

In this study, we use *LbNOX* to dissect the roles of oxidation in CAR T-cells, differentiating oxidative ATP production (unaffected by *LbNOX*) from total NADH oxidation (enhanced by *LbNOX*). We found that *LbNOX* conferred several metabolic benefits to CAR T-cells, including an increased ability to oxidize lactate, an enhanced ability to regenerate intracellular NAD^+^, and increased resistance to ETC disruption. Despite these metabolic attributes, results from our xenograft model revealed that a subset of *LbNOX*-expressing CAR T-cells had an inferior ability to eradicate tumors relative to control (Figure 5D–F). Targeting reductive stress alone is an ineffective approach to enhance adoptive immunotherapies in solid tumors. By distinguishing the relative importance of reductive stress versus ATP production in our model, our findings implicate energy deficits as a critical barrier to CAR T-cell immunotherapies in hypoxic tumors.

Strategies to enhance T-cell metabolic fitness have shown promise in a number of preclinical models. Limiting Warburg metabolism by inhibiting hexokinase [20], LDH [21], P38MAPK [22], arginine conditioning [23], and glucose restriction [24] improves the metabolic, phenotypic, and functional features of cultured T-cells. An inherent limitation shared by these approaches is that they are confined to the ex vivo expansion phase prior to adoptive transfer. Developing approaches to overcome the metabolic nature of the TME requires a deeper understanding of the mechanisms limiting metabolic fitness in situ.

Intra-tumoral hypoxia correlates with T-cell hypofunction and poor response to PD-1 blockade in syngeneic models of melanoma [7]. Restoring access to oxygen rather than glucose enhanced T-cell antitumor function, implicating oxygen as a critical metabolite for tumor-infiltrating lymphocytes. Our data provide mechanistic insight into the oxygen requirements of T-cells traversing solid tumors: the benefits of oxygen may be inextricably tied to its role in ATP replenishment. Future studies may reveal how CAR design influences the effectiveness of *LbNOX* following adoptive transfer. While we focused our attention on CD28 costimulation, the inherent ability of 4-1BB or ICOS to sustain high rates of oxidative phosphorylation, along with their contingency energy reserve, would likely extend CAR T-cell persistence and survival in solid tumors. Alleviating reductive stress (*LbNOX)* in CAR T-cells geared for long-term immunosurveillance, with enhanced energy-generating capacity, may be a better strategy for success against solid tumors.

We show that ^13^C_3_ lactate supports TCA cycle anaplerosis and short chain CoA synthesis in CAR T-cells (Figure 1), challenging the widely-accepted belief that lactate is inherently immunosuppressive [25,26]. In vivo, tumor cells may co-opt select monocarboxylate transporters to restrict access to local lactate pools, sequestering it for their own benefit [27].

To effectively use lactate as fuel instead of glucose, cells require a functional electron transport chain to generate ATP from NADH. Expressing *LbNOX* constitutively may deplete NADH and thus aerobic ATP production. Elevated NAD may also drive enhanced rates of glycolysis during ex vivo expansion; committing CAR T-cells to an effector differentiated program prior to adoptive transfer. We previously showed that short-lived glycolytic effector CAR T-cells have limited therapeutic potential due to poor engraftment and impaired persistence following infusion [1]. By avoiding such in vitro reprogramming, the controlled induction of *LbNOX* after adoptive transfer may have benefits that are not captured in the present constitutive expression experiments.

Taken together, our findings highlight the challenges in developing strategies targeting individual aspects of metabolic dysfunction in solid tumors. The decoupling of NADH oxidation and ATP production was ineffective. Metabolic enhancements that maintain such coupling are future priorities.

## Data Availability

The data presented in this study are available on request from the corresponding author.

## Supplementary Materials

The following are available online at https://www.mdpi.com/article/10.3390/cells10092334/s1, Figure S1: CAR Design, Figure S2: Lactate supports long chain fatty acid synthesis in murine T cells, Figure S3: Nonadditive effect of Lactate Dehydrogenase inhibition (Ldhi) with CAR T-cells in vivo, Video S1: CAR T-cells depend on coupling of NADH oxidation with ATP production.
