# Supplementary material for: CAR T-Cells Depend on the Coupling of NADH Oxidation with ATP Production
Source: Cells. 2021 Sep 6;10(9):2334. doi: 10.3390/cells10092334 (PMC8472053; doi:10.3390/cells10092334)
Supplement: Supplementary file 1 [file cells-10-02334-s001.zip › cells-1328946-supplementary.pdf]

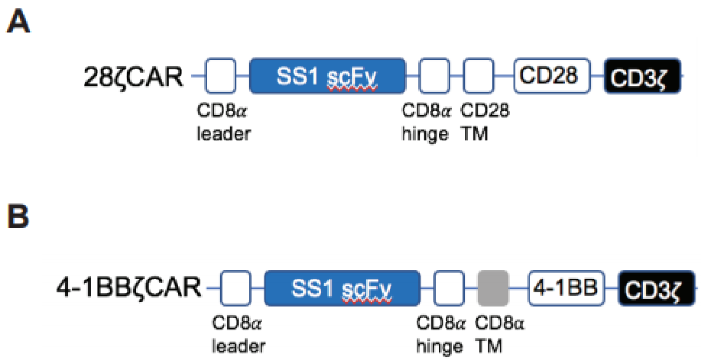

Figure S1. CAR design. (**A**) Schematic representation of the mesothelin-specific 28ζ lentiviral CAR construct containing an SS1 single chain variable fragment (scFv) which is linked via a CD8α hinge as well as a CD28 TM to the CD28 and CD3ζ intracellular signaling domains. TM, transmembrane. (**B**) The 4-1BBζ CAR construct has a CD8α hinge, CD8α TM and 4-1BB intracellular signaling domain.

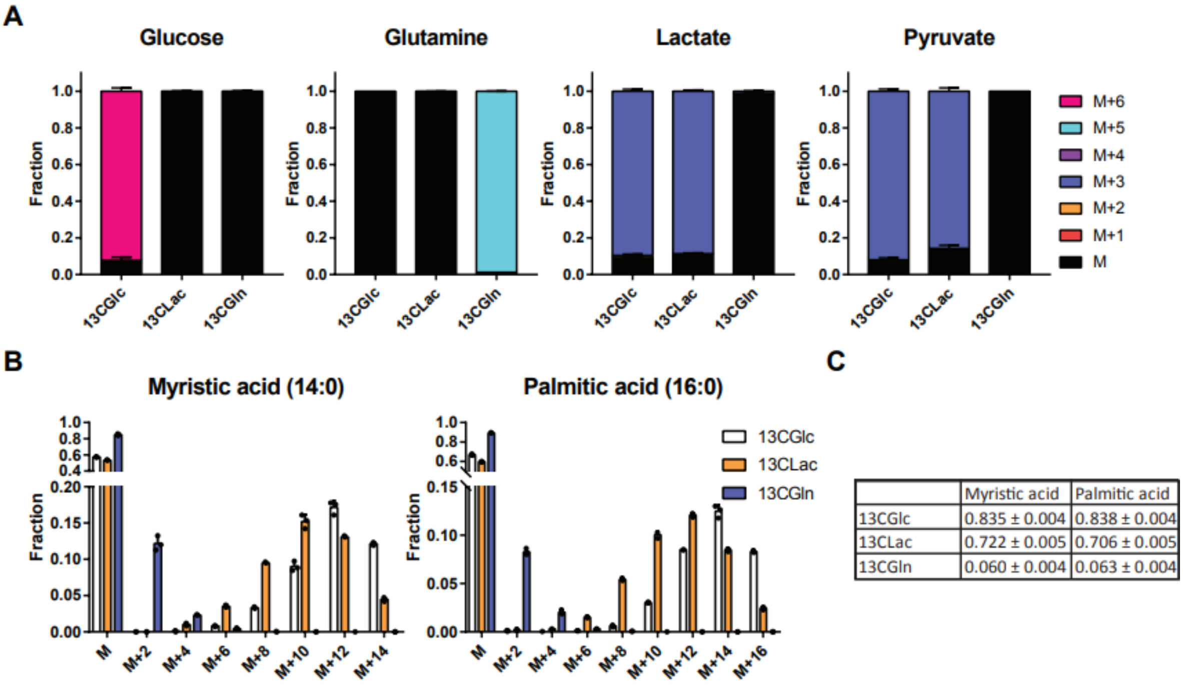

C

	Myristic acid	Palmitic acid
13CGlc	0.835 ± 0.004	0.838 ± 0.004
13CLac	0.722 ± 0.005	0.706 ± 0.005
13CGln	0.060 ± 0.004	0.063 ± 0.004

Figure S2. Lactate metabolism supports long chain fatty acid synthesis in murine T-cells. (**A**) Metabolite labeling patterns in activated CD8+ T-cells (day after a 24 h incubation with U-13-C glucose, U-13-C-lactate, or U-13C-glutamine (mean ± SD, *n* = 3). (**B**) Myristate and palmitate labeling from U-13-C glucose, U-13-C-lactate, or U-13C-glutamine following a 24 h incubation time (mean ± SD, *n* = 3). (**C**) Fractional contribution to myristate and palmitate labeling from U-13-C glucose, U-13-C-lactate, or U-13C-glutamine (mean ± SD, *n* = 3). 13CGlc, U-13-C glucose; 13Clac, U-13-C-lactate; 13CGln, U-13C-glutamine.

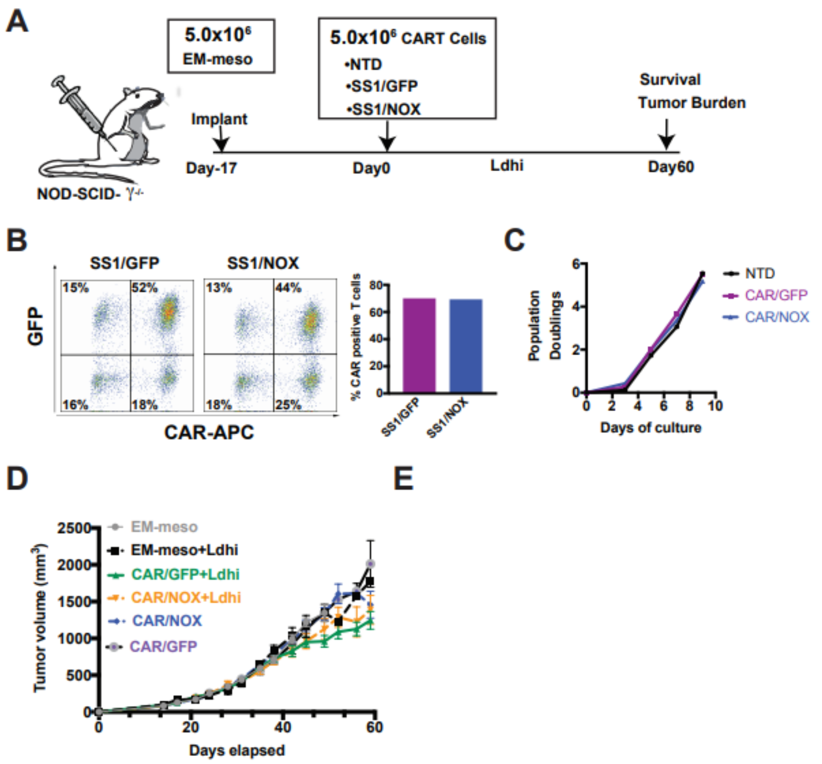

Figure S3. Non-additive effects of Ldhi with CAR T-cell anti-tumor function in vivo.

Experimental layout including a schematic of the EM-meso xenograft model and combinatorial treatment approach. (**A**) 5 × 10^6 EM-meso cells were injected subcutaneously into adult NSG mice. 5 ×10^6 28ζ CAR+ T-cells were I.V. injected 17 days after tumor implantation (n = 6–9 per group). 75mg/kg Ldhi or vehicle (PBS) were administered twice weekly (days 21–49). Tumor volumes were measured by caliper at the indicated time-points as described in the Materials and Methods section. Values represent mean ± S.E.M. for each group. (B&C) Tumor size, independent of CAR cytolytic activity, was compared following Ldhi inhibition. (C&D) The anti-tumor function of CAR T-cells following Ldhi inhibition was compared by Two-way ANOVA using a Holm-Sidak multiple comparison post-hoc test. Tumor size was significantly less (*$p < 0.05$ CAR [n = 7] relative to Tumor alone [n = 7]; CAR +Ldhi [n = 7] relative to Tumor alone) during the terminal phase of the experiment (day 49). However, no differences were observed between CAR vs CAR+Ldhi.
